# (5,7,7,12,14,14-Hexamethyl-1,4,8,11-tetra­aza­cyclo­tetra­deca-4,11-diene)nickel(II) bis­[*O*,*O*′-bis­(4-*tert*-butyl­phen­yl) dithio­phosphate]

**DOI:** 10.1107/S1600536810049615

**Published:** 2010-12-04

**Authors:** Chuan Lai, Bin Xie, Li-Ke Zou, Jian-Shen Feng

**Affiliations:** a College of Material and Chemical Engineering, Sichuan University of Science and Engineering, 643000 Zigong, Sichuan, People’s Republic of China; b Research Institute of Functional Material, Sichuan University of Science and Engineering, 643000 Zigong, Sichuan, People’s Republic of China

## Abstract

The title salt, [Ni(C_16_H_32_N_4_)](C_20_H_26_O_2_PS_2_)_2_, comprises a centrosymmetric [Ni(Me_6_[14]dieneN_4_)]^2+^ dication (Me_6_[14]dieneN_4_ is 5,7,7,12,14,14-hexa­methyl-1,4,8,11-tetra­aza­cyclo­tetra­deca-4,11-diene) and two *O*,*O*′-bis­(4-*tert*-butyl­phen­yl) dithio­phosphate anions. The Ni^II^ ion lies on an inversion centre and displays a slightly distorted NiN_4_ square-planar chelation arrangement with four N atoms from the Me_6_[14]dieneN_4_ macrocycle. Two S atoms from symmetry-related anions are located in pseudo-axial positions with respect to the Ni^II^ ion, with Ni⋯S distances of 3.2991 (7) Å. Inter­molecular N—H⋯S and C—H⋯S hydrogen bonds link the complex cation and pair of anions into a 1:2 type salt.

## Related literature

For synthetic procedures, see: Li & Xie (1997 ▶); Xie *et al.* (2009 ▶). For applications as mimetic enzymes of transition metal complexes of tetra­mine macrocycles, see: Aoki & Kimura (2002 ▶). For related structures, see: Feng *et al.* (2010 ▶); He *et al.* (2010 ▶); Zou *et al.* (2010 ▶).
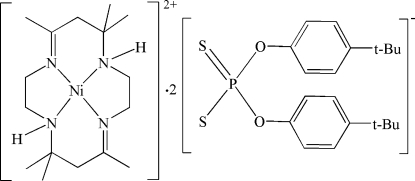


## Experimental

### 

#### Crystal data

[Ni(C_16_H_32_N_4_)](C_20_H_26_O_2_PS_2_)_2_
*M* *_r_* = 1126.16
Triclinic, 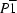

*a* = 9.445 (2) Å
*b* = 12.168 (3) Å
*c* = 12.740 (3) Å
α = 95.965 (4)°
β = 91.360 (3)°
γ = 99.787 (4)°
*V* = 1433.7 (5) Å^3^
*Z* = 1
Mo *K*α radiation
μ = 0.59 mm^−1^
*T* = 103 K
0.27 × 0.23 × 0.08 mm

#### Data collection

Rigaku SPIDER diffractometer
Absorption correction: multi-scan (*ABSCOR*; Higashi, 1995 ▶) *T*_min_ = 0.859, *T*_max_ = 0.955
14032 measured reflections
6434 independent reflections
4838 reflections with *I* > 2σ(*I*)
*R*_int_ = 0.036

#### Refinement

*R*[*F* ^2^ > 2σ(*F* ^2^)] = 0.041
*wR*(*F* ^2^) = 0.101
*S* = 1.00
6434 reflections
335 parameters
H atoms treated by a mixture of independent and constrained refinement
Δρ_max_ = 0.36 e Å^−3^
Δρ_min_ = −0.35 e Å^−3^

### 

Data collection: *RAPID-AUTO* (Rigaku, 2004 ▶); cell refinement: *RAPID-AUTO*; data reduction: *RAPID-AUTO*; program(s) used to solve structure: *SHELXS97* (Sheldrick, 2008 ▶); program(s) used to refine structure: *SHELXL97* (Sheldrick, 2008 ▶); molecular graphics: *ORTEP-3 for Windows* (Farrugia, 1997 ▶); software used to prepare material for publication: *SHELXL97*.

## Supplementary Material

Crystal structure: contains datablocks I, global. DOI: 10.1107/S1600536810049615/pv2360sup1.cif

Structure factors: contains datablocks I. DOI: 10.1107/S1600536810049615/pv2360Isup2.hkl

Additional supplementary materials:  crystallographic information; 3D view; checkCIF report

## Figures and Tables

**Table 1:** Hydrogen-bond geometry (Å, °)

*D*—H⋯*A*	*D*—H	H⋯*A*	*D*⋯*A*	*D*—H⋯*A*
N2—H2*N*⋯S2^i^	0.88 (3)	2.70 (3)	3.542 (2)	162 (3)
C7—H7*A*⋯S2^i^	0.98	2.82	3.703 (3)	150

Symmetry code: (i) 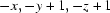.

**Fig. 1.**
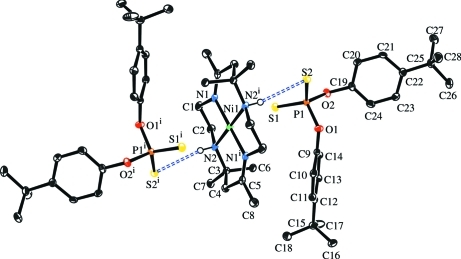
The molecular structure of the title complex, showing the atom-numbering scheme with displacement ellipsoids at 50% probability level. H atoms on N are represented as small spheres of arbitary radii and H atoms on C have been omitted for the sake of clarity. Atoms with the superscript i are generated by the symmetry operation (-x, -y + 1,-z + 1).

## supplementary crystallographic information

### Comment

The synthesis and structural investigation of tetramine macrocycle have attracted much attention due to their analogy to naturally occurring macrocyclic systems and the potential applications as mimetic enzymes of their transition metal complexes (Aoki *et al.*, 2002). We have recently reported the crystal structures of tetramine macrocyclic transition metal adducts with *O*,*O*'-dialkyldithiophosphate (He *et al.*, 2010; Feng *et al.*, 2010; Zou *et al.*, 2010). We report herein the synthesis and structure of an analogous adduct, [Ni(Me_6_[14]dieneN_4_)][S_2_P(OC_6_H_4_Me-4)_2_]_2_, where Me_6_[14]dieneN_4_ is 5,7,7,12,14,14-hexamethyl-1,4,8,11-tetraazacyclotetradeca-4,11-diene.

In the centrosymmetric structure of the title adduct, Ni^II^ ion lies on an inversion centre and is accommodated in the tetradentatic 14-membered tetramine macrocycle cavity in a slightly distorted mononuclear NiN_4_ square-planar geometry (Fig. 1). The net charge on Ni^II^ ion is balanced by two symmetry related *O*,*O*'- bis(4-*tert*-butylphenyl)dithiophosphate anions, which are located in the pseudo-axial positions with respect to the inversion centre with Ni···S distances of 3.2991 (7) Å. The complex cations and anions interact with each other through intermolecular N—H···S and C—H···S hydrogen bonds (Table 1).

### Experimental

[Et_2_NH_2_][S_2_P(OC_6_H_4_(Bu-t)-4)_2_] was synthesized according to the procedure described by Li & Xie (1997). [Ni(Me_6_[14]dieneN_4_)](ClO_4_)_2_ was prepared by the method reported by Xie *et al.* (2009). The title adduct was obtained by the reaction of [Ni(Me_6_[14]dieneN_4_)](ClO_4_)_2_ (0.541 g, 1 mmol) and [Et_2_NH_2_][S_2_P(OC_6_H_4_(Bu-t)-4)_2_] (0.936 g, 2 mmol) in refluxing methanol for 3 h. After cooling to room temperature, the precipitate was filtered off, washed with diethyl ether and recrystallized from benzene. The obtained solid was dissolved in hot methanol and filtered, the filtrate was slowly evaporated at room temperature for several days until the formation of orange platelet crystals of the title adduct.

### Refinement

H atoms on C atoms were fixed geometrically and treated as riding, with C—H = 0.99Å (methylene), 0.98Å (methyl), 0.95 Å(aromatic) and *U*_iso_(H) = 1.2*U*_eq_(C). The H atom on N atom was determined from a difference Fourier synthesis and refined isotropically.

### Crystal data

**Table:**

[Ni(C_16_H_32_N_4_)](C_20_H_26_O_2_PS_2_)_2_	*Z* = 1
*M**_r_* = 1126.16	*F*(000) = 602
Triclinic, *P*1	*D*_x_ = 1.304 Mg m^−^^3^
Hall symbol: -P 1	Mo *K*α radiation, λ = 0.71073 Å
*a* = 9.445 (2) Å	Cell parameters from 3818 reflections
*b* = 12.168 (3) Å	θ = 3.0–27.5°
*c* = 12.740 (3) Å	µ = 0.59 mm^−^^1^
α = 95.965 (4)°	*T* = 103 K
β = 91.360 (3)°	Plate, orange
γ = 99.787 (4)°	0.27 × 0.23 × 0.08 mm
*V* = 1433.7 (5) Å^3^	

### Data collection

**Table:**

Rigaku SPIDER diffractometer	6434 independent reflections
Radiation source: Rotating Anode	4838 reflections with *I* > 2σ(*I*)
graphite	*R*_int_ = 0.036
ω scans	θ_max_ = 27.5°, θ_min_ = 3.0°
Absorption correction: multi-scan (*ABSCOR*; Higashi, 1995)	*h* = −12→12
*T*_min_ = 0.859, *T*_max_ = 0.955	*k* = −15→14
14032 measured reflections	*l* = −16→16

### Refinement

**Table:**

Refinement on *F*^2^	Primary atom site location: structure-invariant direct methods
Least-squares matrix: full	Secondary atom site location: difference Fourier map
*R*[*F*^2^ > 2σ(*F*^2^)] = 0.041	Hydrogen site location: inferred from neighbouring sites
*wR*(*F*^2^) = 0.101	H atoms treated by a mixture of independent and constrained refinement
*S* = 1.00	*w* = 1/[σ^2^(*F*_o_^2^) + (0.0451*P*)^2^ + 0.698*P*] where *P* = (*F*_o_^2^ + 2*F*_c_^2^)/3
6434 reflections	(Δ/σ)_max_ = 0.001
335 parameters	Δρ_max_ = 0.36 e Å^−^^3^
0 restraints	Δρ_min_ = −0.35 e Å^−^^3^

### Special details

**Table:**

Geometry. All e.s.d.'s (except the e.s.d. in the dihedral angle between two l.s. planes) are estimated using the full covariance matrix. The cell e.s.d.'s are taken into account individually in the estimation of e.s.d.'s in distances, angles and torsion angles; correlations between e.s.d.'s in cell parameters are only used when they are defined by crystal symmetry. An approximate (isotropic) treatment of cell e.s.d.'s is used for estimating e.s.d.'s involving l.s. planes.
Refinement. Refinement of *F*^2^ against ALL reflections. The weighted *R*-factor *wR* and goodness of fit *S* are based on *F*^2^, conventional *R*-factors *R* are based on *F*, with *F* set to zero for negative *F*^2^. The threshold expression of *F*^2^ > σ(*F*^2^) is used only for calculating *R*-factors(gt) *etc*. and is not relevant to the choice of reflections for refinement. *R*-factors based on *F*^2^ are statistically about twice as large as those based on *F*, and *R*- factors based on ALL data will be even larger.

### Fractional atomic coordinates and isotropic or equivalent isotropic displacement parameters (Å^2^)

**Table:**

	*x*	*y*	*z*	*U*_iso_*/*U*_eq_	
Ni1	0.0000	0.5000	0.5000	0.01467 (11)	
P1	0.38240 (6)	0.74491 (5)	0.59769 (5)	0.01291 (13)	
S1	0.18719 (6)	0.75534 (5)	0.55415 (5)	0.01884 (14)	
S2	0.41409 (6)	0.65477 (5)	0.71245 (4)	0.01634 (13)	
O1	0.47162 (17)	0.69494 (13)	0.50147 (12)	0.0163 (3)	
O2	0.46883 (16)	0.87255 (13)	0.62254 (12)	0.0161 (3)	
N1	−0.11781 (19)	0.56640 (16)	0.59937 (15)	0.0147 (4)	
N2	−0.1166 (2)	0.54850 (17)	0.39160 (15)	0.0147 (4)	
C1	−0.2353 (2)	0.6077 (2)	0.54470 (19)	0.0186 (5)	
H1A	−0.2638	0.6718	0.5885	0.022*	
H1B	−0.3203	0.5474	0.5312	0.022*	
C2	−0.1784 (2)	0.6437 (2)	0.44242 (18)	0.0180 (5)	
H2A	−0.2568	0.6602	0.3969	0.022*	
H2B	−0.1036	0.7118	0.4555	0.022*	
C3	−0.0567 (2)	0.56507 (19)	0.28493 (18)	0.0158 (5)	
C4	−0.0187 (2)	0.4525 (2)	0.24158 (18)	0.0180 (5)	
H4A	−0.1058	0.3944	0.2416	0.022*	
H4B	0.0075	0.4569	0.1672	0.022*	
C5	0.1014 (2)	0.41448 (19)	0.29968 (19)	0.0168 (5)	
C6	0.0743 (2)	0.6583 (2)	0.29262 (18)	0.0172 (5)	
H6A	0.0457	0.7298	0.3179	0.021*	
H6B	0.1147	0.6633	0.2228	0.021*	
H6C	0.1468	0.6417	0.3421	0.021*	
C7	−0.1720 (2)	0.5932 (2)	0.20931 (18)	0.0176 (5)	
H7A	−0.2588	0.5359	0.2077	0.021*	
H7B	−0.1351	0.5950	0.1381	0.021*	
H7C	−0.1954	0.6668	0.2341	0.021*	
C8	0.1938 (3)	0.3504 (2)	0.2313 (2)	0.0233 (5)	
H8A	0.2953	0.3819	0.2480	0.028*	
H8B	0.1710	0.3562	0.1568	0.028*	
H8C	0.1753	0.2714	0.2444	0.028*	
C9	0.4542 (2)	0.70913 (19)	0.39500 (17)	0.0135 (4)	
C10	0.4612 (2)	0.61550 (19)	0.32408 (18)	0.0148 (5)	
H10	0.4730	0.5466	0.3492	0.018*	
C11	0.4510 (2)	0.62328 (19)	0.21665 (18)	0.0153 (5)	
H11	0.4557	0.5590	0.1685	0.018*	
C12	0.4338 (2)	0.72341 (19)	0.17723 (18)	0.0155 (5)	
C13	0.4277 (2)	0.81515 (19)	0.25037 (18)	0.0167 (5)	
H13	0.4161	0.8842	0.2256	0.020*	
C14	0.4379 (2)	0.80954 (19)	0.35900 (18)	0.0165 (5)	
H14	0.4337	0.8737	0.4074	0.020*	
C15	0.4216 (3)	0.7279 (2)	0.05768 (18)	0.0190 (5)	
C16	0.5521 (3)	0.6889 (2)	0.00546 (19)	0.0248 (6)	
H16A	0.5442	0.6924	−0.0709	0.030*	
H16B	0.5550	0.6115	0.0192	0.030*	
H16C	0.6404	0.7379	0.0348	0.030*	
C17	0.4152 (3)	0.8452 (2)	0.0292 (2)	0.0342 (7)	
H17A	0.4071	0.8437	−0.0478	0.041*	
H17B	0.5030	0.8962	0.0564	0.041*	
H17C	0.3315	0.8712	0.0606	0.041*	
C18	0.2848 (3)	0.6490 (3)	0.0122 (2)	0.0341 (7)	
H18A	0.2004	0.6748	0.0428	0.041*	
H18B	0.2877	0.5728	0.0295	0.041*	
H18C	0.2790	0.6491	−0.0647	0.041*	
C19	0.6129 (2)	0.89734 (18)	0.65979 (18)	0.0155 (5)	
C20	0.6442 (2)	0.90526 (19)	0.76688 (18)	0.0151 (5)	
H20	0.5700	0.8875	0.8145	0.018*	
C21	0.7862 (2)	0.93967 (19)	0.80414 (18)	0.0162 (5)	
H21	0.8080	0.9446	0.8778	0.019*	
C22	0.8978 (2)	0.96726 (19)	0.73619 (18)	0.0167 (5)	
C23	0.8612 (3)	0.9561 (2)	0.62884 (19)	0.0220 (5)	
H23	0.9349	0.9724	0.5804	0.026*	
C24	0.7195 (3)	0.9215 (2)	0.59016 (19)	0.0208 (5)	
H24	0.6970	0.9148	0.5164	0.025*	
C25	1.0515 (2)	1.0085 (2)	0.78141 (19)	0.0192 (5)	
C26	1.1533 (3)	1.0529 (2)	0.6975 (2)	0.0265 (6)	
H26A	1.1627	0.9913	0.6436	0.032*	
H26B	1.2479	1.0848	0.7308	0.032*	
H26C	1.1142	1.1111	0.6643	0.032*	
C27	1.0506 (3)	1.1031 (2)	0.8708 (2)	0.0261 (6)	
H27A	1.0100	1.1640	0.8436	0.031*	
H27B	1.1492	1.1317	0.8984	0.031*	
H27C	0.9919	1.0741	0.9276	0.031*	
C28	1.1095 (3)	0.9114 (2)	0.8255 (2)	0.0258 (6)	
H28A	1.0468	0.8826	0.8803	0.031*	
H28B	1.2070	0.9383	0.8560	0.031*	
H28C	1.1117	0.8513	0.7684	0.031*	
H2N	−0.185 (3)	0.490 (3)	0.379 (2)	0.046 (10)*	

### Atomic displacement parameters (Å^2^)

**Table:**

	*U*^11^	*U*^22^	*U*^33^	*U*^12^	*U*^13^	*U*^23^
Ni1	0.0110 (2)	0.0202 (2)	0.0153 (2)	0.00699 (16)	0.00251 (16)	0.00626 (17)
P1	0.0122 (3)	0.0134 (3)	0.0131 (3)	0.0027 (2)	0.0002 (2)	0.0005 (2)
S1	0.0125 (3)	0.0244 (3)	0.0195 (3)	0.0046 (2)	−0.0016 (2)	0.0007 (2)
S2	0.0169 (3)	0.0166 (3)	0.0154 (3)	0.0011 (2)	−0.0002 (2)	0.0047 (2)
O1	0.0179 (8)	0.0211 (9)	0.0119 (8)	0.0091 (6)	0.0002 (6)	0.0024 (7)
O2	0.0155 (8)	0.0124 (8)	0.0199 (9)	0.0017 (6)	−0.0031 (7)	0.0018 (7)
N1	0.0100 (9)	0.0168 (10)	0.0182 (10)	0.0036 (7)	0.0015 (7)	0.0045 (8)
N2	0.0113 (9)	0.0162 (10)	0.0185 (10)	0.0045 (7)	0.0029 (8)	0.0064 (8)
C1	0.0118 (11)	0.0243 (13)	0.0225 (13)	0.0089 (9)	0.0019 (9)	0.0062 (10)
C2	0.0159 (11)	0.0198 (12)	0.0204 (12)	0.0089 (9)	0.0018 (9)	0.0033 (10)
C3	0.0126 (11)	0.0187 (12)	0.0169 (12)	0.0027 (8)	0.0019 (9)	0.0059 (9)
C4	0.0148 (11)	0.0220 (12)	0.0167 (12)	0.0028 (9)	−0.0010 (9)	0.0012 (10)
C5	0.0113 (11)	0.0167 (12)	0.0217 (12)	0.0002 (8)	0.0015 (9)	0.0031 (10)
C6	0.0137 (11)	0.0225 (12)	0.0157 (12)	0.0017 (9)	0.0006 (9)	0.0055 (10)
C7	0.0144 (11)	0.0211 (12)	0.0184 (12)	0.0038 (9)	−0.0025 (9)	0.0074 (10)
C8	0.0204 (13)	0.0244 (13)	0.0251 (13)	0.0080 (10)	−0.0006 (10)	−0.0040 (11)
C9	0.0095 (10)	0.0197 (12)	0.0111 (11)	0.0022 (8)	0.0003 (8)	0.0016 (9)
C10	0.0122 (11)	0.0139 (11)	0.0185 (12)	0.0031 (8)	0.0022 (9)	0.0020 (9)
C11	0.0131 (11)	0.0153 (11)	0.0174 (12)	0.0034 (8)	0.0014 (9)	−0.0009 (9)
C12	0.0099 (10)	0.0211 (12)	0.0153 (11)	0.0026 (8)	0.0005 (8)	0.0011 (9)
C13	0.0186 (12)	0.0150 (11)	0.0168 (12)	0.0028 (9)	0.0005 (9)	0.0026 (9)
C14	0.0154 (11)	0.0167 (12)	0.0167 (12)	0.0023 (9)	−0.0008 (9)	−0.0005 (9)
C15	0.0185 (12)	0.0232 (13)	0.0157 (12)	0.0047 (9)	0.0004 (9)	0.0029 (10)
C16	0.0221 (13)	0.0367 (16)	0.0158 (12)	0.0057 (11)	0.0045 (10)	0.0017 (11)
C17	0.056 (2)	0.0365 (17)	0.0163 (13)	0.0209 (14)	0.0063 (13)	0.0094 (12)
C18	0.0226 (14)	0.060 (2)	0.0152 (13)	−0.0037 (13)	−0.0026 (11)	0.0036 (13)
C19	0.0134 (11)	0.0118 (11)	0.0208 (12)	0.0013 (8)	−0.0024 (9)	0.0013 (9)
C20	0.0175 (11)	0.0139 (11)	0.0141 (11)	0.0016 (8)	0.0041 (9)	0.0026 (9)
C21	0.0201 (12)	0.0180 (12)	0.0093 (11)	0.0007 (9)	0.0002 (9)	0.0004 (9)
C22	0.0162 (11)	0.0143 (11)	0.0189 (12)	−0.0003 (8)	0.0008 (9)	0.0036 (9)
C23	0.0187 (12)	0.0285 (14)	0.0164 (12)	−0.0029 (10)	0.0044 (10)	0.0019 (10)
C24	0.0231 (13)	0.0241 (13)	0.0135 (12)	−0.0012 (10)	−0.0013 (10)	0.0027 (10)
C25	0.0142 (11)	0.0217 (13)	0.0203 (12)	−0.0019 (9)	−0.0006 (9)	0.0039 (10)
C26	0.0156 (12)	0.0319 (15)	0.0289 (14)	−0.0044 (10)	0.0004 (10)	0.0033 (12)
C27	0.0246 (13)	0.0222 (14)	0.0261 (14)	−0.0069 (10)	−0.0025 (11)	−0.0036 (11)
C28	0.0201 (13)	0.0314 (15)	0.0266 (14)	0.0054 (10)	−0.0015 (11)	0.0061 (11)

### Geometric parameters (Å, °)

**Table:**

Ni1—N1	1.9114 (19)	C11—C12	1.398 (3)
Ni1—N1^i^	1.9114 (19)	C11—H11	0.9500
Ni1—N2	1.9457 (19)	C12—C13	1.388 (3)
Ni1—N2^i^	1.9457 (19)	C12—C15	1.532 (3)
Ni1—S1	3.2990 (9)	C13—C14	1.394 (3)
P1—O2	1.6226 (16)	C13—H13	0.9500
P1—O1	1.6281 (17)	C14—H14	0.9500
P1—S1	1.9413 (9)	C15—C17	1.519 (4)
P1—S2	1.9632 (9)	C15—C18	1.534 (3)
O1—C9	1.394 (3)	C15—C16	1.536 (3)
O2—C19	1.403 (3)	C16—H16A	0.9800
N1—C5^i^	1.284 (3)	C16—H16B	0.9800
N1—C1	1.481 (3)	C16—H16C	0.9800
N2—C2	1.481 (3)	C17—H17A	0.9800
N2—C3	1.504 (3)	C17—H17B	0.9800
N2—H2N	0.88 (3)	C17—H17C	0.9800
C1—C2	1.500 (3)	C18—H18A	0.9800
C1—H1A	0.9900	C18—H18B	0.9800
C1—H1B	0.9900	C18—H18C	0.9800
C2—H2A	0.9900	C19—C24	1.372 (3)
C2—H2B	0.9900	C19—C20	1.379 (3)
C3—C6	1.524 (3)	C20—C21	1.392 (3)
C3—C4	1.526 (3)	C20—H20	0.9500
C3—C7	1.542 (3)	C21—C22	1.399 (3)
C4—C5	1.503 (3)	C21—H21	0.9500
C4—H4A	0.9900	C22—C23	1.390 (3)
C4—H4B	0.9900	C22—C25	1.531 (3)
C5—N1^i^	1.284 (3)	C23—C24	1.395 (3)
C5—C8	1.501 (3)	C23—H23	0.9500
C6—H6A	0.9800	C24—H24	0.9500
C6—H6B	0.9800	C25—C26	1.534 (3)
C6—H6C	0.9800	C25—C28	1.534 (4)
C7—H7A	0.9800	C25—C27	1.535 (3)
C7—H7B	0.9800	C26—H26A	0.9800
C7—H7C	0.9800	C26—H26B	0.9800
C8—H8A	0.9800	C26—H26C	0.9800
C8—H8B	0.9800	C27—H27A	0.9800
C8—H8C	0.9800	C27—H27B	0.9800
C9—C14	1.380 (3)	C27—H27C	0.9800
C9—C10	1.390 (3)	C28—H28A	0.9800
C10—C11	1.384 (3)	C28—H28B	0.9800
C10—H10	0.9500	C28—H28C	0.9800
			
N1—Ni1—N1^i^	180.0	C10—C11—C12	121.6 (2)
N1—Ni1—N2	86.13 (8)	C10—C11—H11	119.2
N1^i^—Ni1—N2	93.87 (8)	C12—C11—H11	119.2
N1—Ni1—N2^i^	93.87 (8)	C13—C12—C11	117.2 (2)
N1^i^—Ni1—N2^i^	86.13 (8)	C13—C12—C15	123.0 (2)
N2—Ni1—N2^i^	180.0	C11—C12—C15	119.8 (2)
N1—Ni1—S1	78.48 (6)	C12—C13—C14	122.3 (2)
N1^i^—Ni1—S1	101.52 (6)	C12—C13—H13	118.8
N2—Ni1—S1	91.63 (6)	C14—C13—H13	118.8
N2^i^—Ni1—S1	88.37 (6)	C9—C14—C13	118.8 (2)
O2—P1—O1	101.95 (9)	C9—C14—H14	120.6
O2—P1—S1	106.69 (7)	C13—C14—H14	120.6
O1—P1—S1	112.73 (6)	C17—C15—C12	112.5 (2)
O2—P1—S2	111.23 (6)	C17—C15—C18	108.5 (2)
O1—P1—S2	103.62 (7)	C12—C15—C18	109.3 (2)
S1—P1—S2	119.33 (4)	C17—C15—C16	108.0 (2)
P1—S1—Ni1	109.04 (3)	C12—C15—C16	109.77 (19)
C9—O1—P1	126.04 (15)	C18—C15—C16	108.7 (2)
C19—O2—P1	122.48 (14)	C15—C16—H16A	109.5
C5^i^—N1—C1	119.43 (19)	C15—C16—H16B	109.5
C5^i^—N1—Ni1	129.37 (16)	H16A—C16—H16B	109.5
C1—N1—Ni1	110.98 (14)	C15—C16—H16C	109.5
C2—N2—C3	115.10 (18)	H16A—C16—H16C	109.5
C2—N2—Ni1	106.77 (14)	H16B—C16—H16C	109.5
C3—N2—Ni1	119.71 (14)	C15—C17—H17A	109.5
C2—N2—H2N	109 (2)	C15—C17—H17B	109.5
C3—N2—H2N	104 (2)	H17A—C17—H17B	109.5
Ni1—N2—H2N	101 (2)	C15—C17—H17C	109.5
N1—C1—C2	106.80 (18)	H17A—C17—H17C	109.5
N1—C1—H1A	110.4	H17B—C17—H17C	109.5
C2—C1—H1A	110.4	C15—C18—H18A	109.5
N1—C1—H1B	110.4	C15—C18—H18B	109.5
C2—C1—H1B	110.4	H18A—C18—H18B	109.5
H1A—C1—H1B	108.6	C15—C18—H18C	109.5
N2—C2—C1	105.96 (19)	H18A—C18—H18C	109.5
N2—C2—H2A	110.5	H18B—C18—H18C	109.5
C1—C2—H2A	110.5	C24—C19—C20	121.0 (2)
N2—C2—H2B	110.5	C24—C19—O2	119.5 (2)
C1—C2—H2B	110.5	C20—C19—O2	119.3 (2)
H2A—C2—H2B	108.7	C19—C20—C21	119.0 (2)
N2—C3—C6	111.51 (19)	C19—C20—H20	120.5
N2—C3—C4	106.03 (19)	C21—C20—H20	120.5
C6—C3—C4	111.42 (19)	C20—C21—C22	121.9 (2)
N2—C3—C7	110.55 (18)	C20—C21—H21	119.1
C6—C3—C7	109.24 (19)	C22—C21—H21	119.1
C4—C3—C7	107.99 (18)	C23—C22—C21	116.9 (2)
C5—C4—C3	116.19 (19)	C23—C22—C25	123.1 (2)
C5—C4—H4A	108.2	C21—C22—C25	119.9 (2)
C3—C4—H4A	108.2	C22—C23—C24	121.9 (2)
C5—C4—H4B	108.2	C22—C23—H23	119.1
C3—C4—H4B	108.2	C24—C23—H23	119.1
H4A—C4—H4B	107.4	C19—C24—C23	119.3 (2)
N1^i^—C5—C8	124.0 (2)	C19—C24—H24	120.4
N1^i^—C5—C4	121.0 (2)	C23—C24—H24	120.4
C8—C5—C4	114.9 (2)	C22—C25—C26	112.07 (19)
C3—C6—H6A	109.5	C22—C25—C28	109.5 (2)
C3—C6—H6B	109.5	C26—C25—C28	108.1 (2)
H6A—C6—H6B	109.5	C22—C25—C27	109.1 (2)
C3—C6—H6C	109.5	C26—C25—C27	108.7 (2)
H6A—C6—H6C	109.5	C28—C25—C27	109.3 (2)
H6B—C6—H6C	109.5	C25—C26—H26A	109.5
C3—C7—H7A	109.5	C25—C26—H26B	109.5
C3—C7—H7B	109.5	H26A—C26—H26B	109.5
H7A—C7—H7B	109.5	C25—C26—H26C	109.5
C3—C7—H7C	109.5	H26A—C26—H26C	109.5
H7A—C7—H7C	109.5	H26B—C26—H26C	109.5
H7B—C7—H7C	109.5	C25—C27—H27A	109.5
C5—C8—H8A	109.5	C25—C27—H27B	109.5
C5—C8—H8B	109.5	H27A—C27—H27B	109.5
H8A—C8—H8B	109.5	C25—C27—H27C	109.5
C5—C8—H8C	109.5	H27A—C27—H27C	109.5
H8A—C8—H8C	109.5	H27B—C27—H27C	109.5
H8B—C8—H8C	109.5	C25—C28—H28A	109.5
C14—C9—C10	120.5 (2)	C25—C28—H28B	109.5
C14—C9—O1	123.8 (2)	H28A—C28—H28B	109.5
C10—C9—O1	115.6 (2)	C25—C28—H28C	109.5
C11—C10—C9	119.6 (2)	H28A—C28—H28C	109.5
C11—C10—H10	120.2	H28B—C28—H28C	109.5
C9—C10—H10	120.2		
			
O2—P1—S1—Ni1	179.72 (6)	C3—C4—C5—N1^i^	36.6 (3)
O1—P1—S1—Ni1	68.62 (7)	C3—C4—C5—C8	−145.5 (2)
S2—P1—S1—Ni1	−53.26 (5)	P1—O1—C9—C14	42.9 (3)
N1—Ni1—S1—P1	126.44 (7)	P1—O1—C9—C10	−140.20 (18)
N1^i^—Ni1—S1—P1	−53.56 (7)	C14—C9—C10—C11	−0.3 (3)
N2—Ni1—S1—P1	−147.85 (6)	O1—C9—C10—C11	−177.36 (19)
N2^i^—Ni1—S1—P1	32.15 (6)	C9—C10—C11—C12	0.1 (3)
O2—P1—O1—C9	−82.29 (18)	C10—C11—C12—C13	0.1 (3)
S1—P1—O1—C9	31.73 (19)	C10—C11—C12—C15	−179.4 (2)
S2—P1—O1—C9	162.11 (15)	C11—C12—C13—C14	0.0 (3)
O1—P1—O2—C19	−63.81 (18)	C15—C12—C13—C14	179.5 (2)
S1—P1—O2—C19	177.78 (15)	C10—C9—C14—C13	0.4 (3)
S2—P1—O2—C19	46.09 (18)	O1—C9—C14—C13	177.2 (2)
N2—Ni1—N1—C5^i^	−169.7 (2)	C12—C13—C14—C9	−0.2 (3)
N2^i^—Ni1—N1—C5^i^	10.3 (2)	C13—C12—C15—C17	4.9 (3)
S1—Ni1—N1—C5^i^	−77.3 (2)	C11—C12—C15—C17	−175.5 (2)
N2—Ni1—N1—C1	4.72 (15)	C13—C12—C15—C18	−115.6 (3)
N2^i^—Ni1—N1—C1	−175.28 (15)	C11—C12—C15—C18	63.9 (3)
S1—Ni1—N1—C1	97.18 (14)	C13—C12—C15—C16	125.3 (2)
N1—Ni1—N2—C2	23.67 (14)	C11—C12—C15—C16	−55.2 (3)
N1^i^—Ni1—N2—C2	−156.33 (14)	P1—O2—C19—C24	98.6 (2)
S1—Ni1—N2—C2	−54.67 (14)	P1—O2—C19—C20	−86.2 (2)
N1—Ni1—N2—C3	156.67 (17)	C24—C19—C20—C21	0.8 (4)
N1^i^—Ni1—N2—C3	−23.33 (17)	O2—C19—C20—C21	−174.3 (2)
S1—Ni1—N2—C3	78.33 (16)	C19—C20—C21—C22	0.5 (4)
C5^i^—N1—C1—C2	143.3 (2)	C20—C21—C22—C23	−1.6 (4)
Ni1—N1—C1—C2	−31.7 (2)	C20—C21—C22—C25	177.9 (2)
C3—N2—C2—C1	178.12 (18)	C21—C22—C23—C24	1.5 (4)
Ni1—N2—C2—C1	−46.42 (19)	C25—C22—C23—C24	−178.0 (2)
N1—C1—C2—N2	50.7 (2)	C20—C19—C24—C23	−0.9 (4)
C2—N2—C3—C6	66.1 (2)	O2—C19—C24—C23	174.2 (2)
Ni1—N2—C3—C6	−63.2 (2)	C22—C23—C24—C19	−0.3 (4)
C2—N2—C3—C4	−172.42 (17)	C23—C22—C25—C26	9.1 (3)
Ni1—N2—C3—C4	58.2 (2)	C21—C22—C25—C26	−170.4 (2)
C2—N2—C3—C7	−55.6 (2)	C23—C22—C25—C28	−110.9 (3)
Ni1—N2—C3—C7	175.03 (14)	C21—C22—C25—C28	69.6 (3)
N2—C3—C4—C5	−66.3 (2)	C23—C22—C25—C27	129.5 (3)
C6—C3—C4—C5	55.2 (3)	C21—C22—C25—C27	−50.0 (3)
C7—C3—C4—C5	175.17 (19)		

Symmetry codes: (i) −*x*, −*y*+1, −*z*+1.

### Hydrogen-bond geometry (Å, °)

**Table:**

*D*—H···*A*	*D*—H	H···*A*	*D*···*A*	*D*—H···*A*
N2—H2N···S2^i^	0.88 (3)	2.70 (3)	3.542 (2)	162 (3)
C7—H7A···S2^i^	0.98	2.82	3.703 (3)	150

Symmetry codes: (i) −*x*, −*y*+1, −*z*+1.
